# The State Medical Society of Pennsylvania

**Published:** 1861-05

**Authors:** 


THE STATE MEDICAL SOCIETY OF PENNSYLVANIA will hold its annual meeting this year in Pittsburg, on the second Wednesday in June. The railroad companies throughout the State will furnish *return tickets*, without charge, to delegates who attend the meeting.

